# The complete mitochondrial genome sequence of *Vanmanenia stenosoma* (Teleostei: Gastromyzontidae)

**DOI:** 10.1080/23802359.2016.1242388

**Published:** 2016-11-12

**Authors:** Xi Chen, Jing Hong Li

**Affiliations:** College of Forestry, South China Agricultural University, Guangzhou, China

**Keywords:** Genome, *Vanmanenia stenosoma*, type species, Zhejiang Province

## Abstract

*Vanmanenia stenosoma* belongs to the order Cypriniformes, and it is a typical torrent loach in China. In this study, we successfully determined the complete mitochondrial genome sequence of *V. stenosoma* (type species of *Vanmanenia*), which is 16,560 base pairs (bp) and contains 13 protein-coding genes, 2 ribosomal RNA genes, 22 transfer RNA genes, and a putative control region. The genome has the same gene order as that found in other species of the family Gastromyzontidae. This suggests those genera share a common ancestral mitogenome. Nevertheless, phylogenetic reconstructions did not support the monophyly of the genus *Vanmanenia.*

*Vanmanenia stenosoma*, a balitorid loach, occurs mainly in Qiantang-jiang and Yong-jiang basin of China. It is rheophilic, living in swift flowing streams and associated rivers in the mountain regions (Boulenger 1901; Chen 1980). In the current study, the sample of *V. stenosoma* (SCAU 0926783) obtained from Lianhuazhen River (29°4′51″N, 119°0′49″E) in Zhejiang Province, China, has been deposited in the collection of South China Agricultural University (SCAU). Total DNA was extracted from alcohol-preserved caudal fin tissue. The entire mitogenome of *V. stenosoma* were determined by the PCR-based approaches. The PCR products were sequenced and analyzed using Ai et al.’s method (Ai et al. 2013).

The complete mitochondrial genome of *V. stenosoma* was 16,560 bp in length (GenBank accession no. KX786161). Similar to other vertebrate mitochondrial genome structure, it was composed of 22 transfer RNA genes, 13 protein-coding genes, 2 ribosomal RNA genes, and a control region.

Most of the genes were encoded on the H-strand except one protein-coding gene (*ND6*) and eight tRNA genes (tRNA-Gln, tRNA-Ala, tRNA-Asn, tRNA-Cys, tRNA-Tyr, tRNA-Ser1 (UGA), tRNA-Glu and tRNA-Pro). The overall base composition of *V. stenosoma* was 28.0% for A, 29.2% for C, 24.8% for T and 18.0% for G, with a higher A + T content of 52.8%. All protein-coding genes shared the initiation codon ATG, except for *CO1* gene, which started with GTG. With the exception of *ND2*, *CO2, ND4* and *Cyt b*, all protein-coding genes were terminated by complete stop codons: TAG was used for *ND1*, *ND3* and *ND5*, TAA was used for *CO1*, *ATP8*, *ATP6*, *CO3*, *ND4L* and *ND6*. The remaining genes, *ND2*, *CO2, ND4* and *Cyt b*, were terminated by incomplete stop codon T. Likewise, numerous balitorid loaches used the similar codon structure to restrain the process of protein translation as well (Chen et al. 2015; Que et al. 2016; Xu & Hu 2016). The longest one was *ND5* gene (1839 bp) in all protein-coding genes, whereas the shortest was *ATP8* gene (168 bp). The two ribosomal RNA genes, 12S rRNA gene (953 bp) and 16S rRNA gene (1680 bp), were located between tRNA-Phe and tRNA-Leu1(UAA) and separated by tRNA-Val. The position of Control region (895 bp) was located between tRNA-Pro and tRNA-Phe.

*Vanmanenia* Hora, 1932 has long been treated as a senior synonym of *Praeformosania* Fang (1935); loaches of the genus *Vanmanenia* are currently represented by ∼20 nominal species in China and Southeast Asia (Chen 1980; Pan et al. 1991; Tang & Chen 2000; Sato et al. 2011; Kottelat 2012; Chen 2013; Yi et al. 2014; Liang et al. 2016). However, based on this study, the molecular phylogeny reveals *V. stenosoma* (type species of *Vanmanenia*) and ‘*V.*’ *pingchowensis* (type species of *Praeformosania*) as separate clades, with the former more closely related to *Formosania lacustris* (type species of *Formosania* Oshima, 1919) than to the latter (Figure 1; the methods returned very similar tree topologies). Such result is qualitatively similar to classification schemes of Fang (1935), in which *Praeformosania* was sister to both *Formosania* and *Vanmanenia*. The *p*-distances between *V. stenosoma* and ‘*V.*’ *pingchowensis* examined here were 18.3% in *CO1*, 13.9% in *Cyt b* and 11.4% in the whole mitogenomes, which were more than those among morphologically distinct species of the same genus within the family Gastromyzontidae. Yet, some molecular phylogenetic analyses did not support the monophyly of *Vanmanenia* (Liu et al. 2012; Tang et al. 2016; Wu et al. 2016). On these grounds, we recommend the exhaustive molecular and morphological studies for clarifying the relationships within or between *Vanmanenia* and *Praeformosania*.

**Figure 1.**
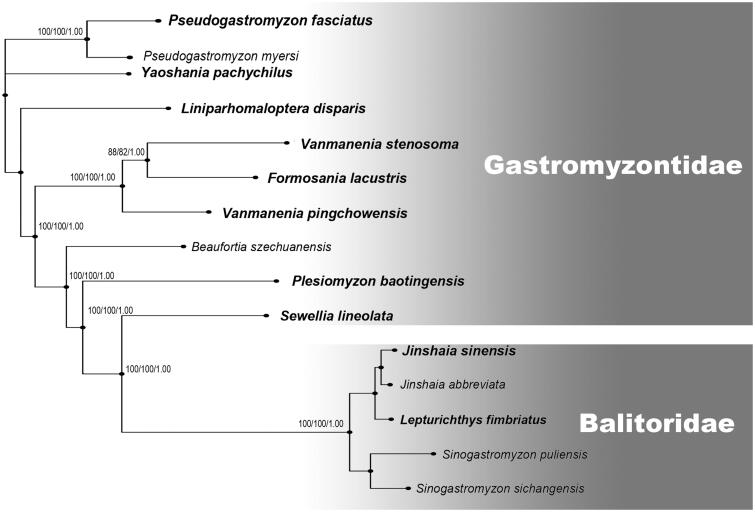
Phylogenetic relationships among *Vanmanenia stenosoma* and other hillstream loaches with complete mitogenome sequences on GenBank were inferred using the Maximum Likelihood (ML), Neighbour-Joining (NJ) and Bayesian (BA) phylogenetic analyses, respectively. Numbers above branches are bootstrap support of the ML, NJ analysis higher than 75 and posterior probabilities of the Bayesian analysis higher than 95%, for the combined analysis. Type species are in bold. The gene’s accession numbers for tree construction are listed as follows: *Pseudogastromyzon fasciatus* (KX101229.1), *Pseudogastromyzon myersi* (AP013300.1), *Yaoshania pachychilus* (KT031050.1), *Liniparhomaloptera disparis* (AP013301.1), *Vanmanenia pingchowensis* (KP005457.1), *Formosania lacustris* (AP010774.1), *Beaufortia szechuanensis* (KP716708.1), *Plesiomyzon baotingensis* (KF732713.1), *Sewellia lineolata* (AP011292.1), *Jinshaia sinensis* (JX155733.1), *Jinshaia abbreviata* (KJ754936.1), *Lepturichthys fimbriata* (KJ830772.1), *Sinogastromyzon sichangensis* (KF711948.1), *Sinogastromyzon puliensis* (FJ605359.1).

## References

[CIT0001] Ai W, Chen X, Xiang D, Chen S, Chen Y. 2013 Coplete mitochondrial genome of *Acrossocheilus wenchowensis* (Cyprinidae, Barbinae). Mitochondrial DNA. 24:249–251. 23305418 10.3109/19401736.2012.752483

[CIT0002] Boulenger GA. 1901 Descriptions of new freshwater fishes discovered by Mr. F. W. Styan at Ningpo, China. Proc Zool Soc London. 1:268–271.

[CIT0003] Chen IS, Wen ZH, Liao CR, Shen CN. 2015 The complete mitochondrial genome of *Plesiomyzon baotingensis* Zheng & Chen (Cyprinifromes, Balitoridae). Mitochondrial DNA. 26:899–901. 24409879 10.3109/19401736.2013.861450

[CIT0004] Chen XY. 2013 Checklist of fishes of Yunnan. Zool Res. 34:281–343. [in Chinese] 10.11813/j.issn.0254-5853.2013.4.0281 23913883

[CIT0005] Chen YY. 1980 Systematic studies of the fishes of the family Homalopteridae of China II. Classification of the fishes of the subfamily Gastromyzoninae. Acta Hydrobiol Sinica. 7:95–120. [in Chinese]

[CIT0006] Fang PW. 1935 Study on the crossostomoid fishes of China. Sinensia. 6:44–97.

[CIT0007] Kottelat M. 2012 Conspectus cobitidum: an inventory of the loaches of the world (Teleostei: Cypriniformes: Cobitoidei). Raffles Bull Zool. 26:1–199.

[CIT0008] Liang ZQ, Wang CR, Wu YA, Li H, Yuan XP, Wei QW. 2016 Complete mitochodrial genome of *Vanmanenia pingchowensis* (Cypriniformes, Cyprinidae). Mitochondrial DNA Part A. 27:2184–2185. 10.3109/19401736.2014.982617 25427810

[CIT0009] Liu SQ, Mayden RL, Zhang JB, Yu D, Tang QY, Deng X, Liu HZ. 2012 Phylogenetic relationships of the Cobitoidea (Teleostei: Cypriniformes) inferred from mitochondrial and nuclear genes with analyses of gene evolution. Gene. 508:60–72. 22868207 10.1016/j.gene.2012.07.040

[CIT0010] Pan JH, Zhong L, Zheng CY, Wu HL, Liu JH. 1991 The freshwater fishes of Guangdong Province. Guangzhou: Guangdong Science and Technology Press p. 267–272. [in Chinese]

[CIT0011] Que YF, Xu DM, Xiong MH, Yang Z, Gao SB, Shi F. 2016 The complete mitochondrial genome of *Jinshaia sinensis* (Teleostei, Balitoridae, Balitorinae). Mitochondrial DNA Part A. 27:949–950. 10.3109/19401736.2014.926500 24937572

[CIT0012] Sato T, Nakajima J, Huang L, Shimatani Y, Hirota SK, Wood C, Kano Y. 2011 Distribution pattern of loaches (Teleostei: Cobitoidea) in the River East Tiaoxi, China. Folia Zool. 60:328.

[CIT0013] Tang QY, Shi LX, Liu F, Yu D, Liu HZ. 2016 Evolution and phylogenetic application of the *MC1R* gene in the Cobitoidea (Teleostei: Cypriniformes). Zool Res. 37:1–11. 10.13918/j.issn.2095-8137.2016.5.281 PMC5071341 27686787

[CIT0014] Tang WQ, Chen YY. 2000 Study on taxonomy of Homalopteridae. J Shanghai Fish Univ. 9:1–9. [in Chinese]

[CIT0015] Wu J, He Y, Ren H, Zhang Y, Du Z, Xie M, Zhu G, Wang Q, Jiang Y, He T, Wen A. 2016 The complete mitochondrial genome sequence of *Beaufortia szechuanensis* (Cypriniformes, Balitoridae). Mitochondrial DNA Part A. 27:2535–2536. 10.3109/19401736.2015.1038792 25922961

[CIT0016] Xu K, Hu F. 2016 The complete mitochondrial genome of *Yaoshania pachychilus* (CHEN, 1980) (Cyprinifromes, Balitoridae). Mitochondrial DNA Part B. 1:207–209. 10.1080/23802359.2016.1155088 PMC7800760 33473453

[CIT0017] Yi WJ, Zhang E, Shen JZ. 2014 *Vanmanenia maculata*, a new species of hillstream loach from the Chang-Jiang Basin, South China (Teleostei: Gastromyzontidae). Zootaxa. 3802:85–97. 10.11646/zootaxa.3802.1.7 24870994

